# Evaluation and Comparison of Vitamin D Responsive Gene Expression in Ovine, Canine and Equine Kidney

**DOI:** 10.1371/journal.pone.0162598

**Published:** 2016-09-15

**Authors:** Sara Azarpeykan, Keren E. Dittmer, Jonathan C. Marshall, Kalyani C. Perera, Erica K. Gee, Els Acke, Keith G. Thompson

**Affiliations:** 1 Institute of Veterinary, Animal and Biomedical Science (IVABS), Massey University, Palmerston North, New Zealand; 2 Institute of Fundamental Sciences (IFS), Massey University, Palmerston North, New Zealand; University of Alabama at Birmingham, UNITED STATES

## Abstract

The aim of this study was to determine the relative abundance and relationship of vitamin D responsive and calcium transporting transcripts (TRPV5, TRPV6, calD_9k_, calD_28k_, PMCA, NCX1, CYP27B1, CYP24A1, and VDR) in ovine, canine and, equine kidney using quantitative real-time PCR (RT-qPCR), and then perform a comparison between the three species. Renal tissue samples were harvested post-mortem from 10 horses, 10 sheep, and five dogs. Primers were designed for each gene. For each sample total RNA was extracted, cDNA synthesised, and RT-qPCR was performed. RT-qPCR data were normalised and statistical comparison was performed. Due to their consistent correlation with each other in each species, TRPV6, calD_9k_/calD_28k_, and PMCA appeared to be the main pathways involved in active transepithelial calcium transport in the kidney of sheep, dogs and horses. The results indicate that all of the studied genes were expressed in the renal tissue of studied species, although the expression levels and correlation of transcripts with each other were different from species to species. All vitamin D responsive and calcium transporting transcripts were highly correlated with VDR in equine kidney, but not in sheep and dogs. The CYP27B1 and CYP24A1 mRNAs showed a different renal expression pattern and correlation in horses compared with sheep and dogs. Given the high urinary calcium concentration and low serum 1,25(OH)_2_D concentration in horses, it could be expected that CYP27B1 expression would be lower than CYP24A1 in the horse, and this did not appear to be the case. The findings suggest that despite low serum vitamin D concentrations, vitamin D still plays a significant role in calcium metabolism in horses, especially given the strong correlations between VDR and vitamin D responsive transcripts in these animals.

## Introduction

In mammals, calcium is the most abundant cation in the body [1]. Calcium participates in many different physiological processes, including neural transmission, muscle contraction, blood coagulation, and mineralisation of bone [2]. The kidney, small intestine, and bone are the three organs that have key roles in the calcium homeostasis system [3].

The kidney plays an important role in calcium homeostasis through the filtration, reabsorption and excretion of calcium [3]. The amount of calcium excreted in the urine is usually low as about 98%–99% of filtered calcium is reabsorbed by the renal tubules [4]. However, increased intestinal calcium absorption or augmented calcium resorption from bone can result in increased plasma calcium concentration leading to increased excretion of calcium in the urine due to compensatory reductions in renal transport capacity [5].

Vitamin D applies its endocrine activity on several calcium-transporting and calcium-sensing tissues to maintain plasma calcium homeostasis [6]. However, vitamin D is biologically inactive and must undergo hydroxylation steps to become activated [7]. In blood, vitamin D binds to vitamin D binding protein (DBP) and is first transported to the liver, where it is hydroxylated to 25-hydroxyvitamin D (25OHD), followed by transport to the kidney where 25OHD undergoes 1α-hydroxylation by cytochrome P450 family 27 subfamily B polypeptide 1 (CYP27B1) resulting in the production of the bioactive form, 1,25-dihydroxyvitamin D (1,25(OH)_2_D) [8]. The kidney also expresses other genes important in vitamin D regulation and calcium homeostasis including the vitamin D receptor (VDR) and cytochrome P450 family 24 subfamily A polypeptide 1 (CYP24A1), the enzyme responsible for the breakdown of 1,25(OH)_2_D [9]. Vitamin D (1,25(OH)_2_D) works as a high affinity ligand for VDR in target tissues and modulates the expression of vitamin D-dependent genes [10].

Calcium is transported across the renal tubule through paracellular and transcellular pathways [2]. Paracellular calcium transport is passive, whereas transcellular calcium transport is active and stimulated by 1,25(OH)_2_D and parathyroid hormone (PTH) [3]. Transcellular calcium transport is regulated by several vitamin D-dependent proteins, including apical influx of calcium by selective calcium channels called the transient receptor potential cation channel subfamily V member 5 (TRPV5) and TRPV6. Calcium diffuses through the cell to the basolateral membrane bound to calcium-binding proteins, such as calbindinD_9k_ (calD_9k_) and calbindinD_28k_ (calD_28k_), and is then secreted across the basolateral membrane via a plasma membrane calcium ATPase (PMCA) and/or sodium calcium exchanger 1 (NCX1) [3, 11].

Species differ considerably in the physiology of their vitamin D and calcium metabolism and there are gaps in the current understanding of calcium transport mechanisms and calcium channels in veterinary science. The aims of the current study were to determine the relative abundance of calcium channels and vitamin D responsive transcripts (TRPV5, TRPV6, calD_9k_, calD_28k_, PMCA, NCX1, CYP27B1, CYP24A1 and, VDR) in ovine, canine and equine kidney using real-time quantitative reverse transcriptase polymerase chain reaction (RT-qPCR), and then to perform a comparison of the expression and correlation of these genes between sheep, dogs and horses in order to determine if the relationships between vitamin D responsive genes were similar between species. In addition, this study aimed to determine if differences in vitamin D responsive gene expression in kidney of horses contributed to the increase urinary excretion of calcium that is a feature of this species.

## Material and Methods

### Sample collection

Kidney samples were harvested post-mortem (Pathobiology Section post-mortem room, Massey University, Palmerston North) from 10 healthy adult horses (five Thoroughbreds, five Standardbreds), 10 healthy adult Romney cross sheep, and five healthy adult mixed breed dogs that were euthanised for reasons unrelated to this study. Tissue samples for RNA isolation were collected within half an hour of euthanasia, cut into small pieces, snap frozen in liquid nitrogen and stored at -80°C until processing. Adjacent samples were collected into 10% neutral buffered formalin and processed for histological examination. Haematoxylin and eosin (H & E) stained sections were examined to confirm the absence of significant lesions.

### RNA extraction and reverse transcription

Total RNA was extracted from renal tissue samples using the RNeasy Mini Kit according to the manufacturer’s instructions (Qiagen, Valencia, California, USA), and included the optional on-column DNase digestion step (RNase-Free DNase Set, Qiagen) during RNA isolation. RNA and DNA concentrations were measured (to check for absence of DNA) using the Qubit® 2.0 Fluorometer with Qubit® RNA HS, and DNA HS Assays (Invitrogen, Life Technologies Corporation, California, USA), and then samples were stored at -80°C until required. The TURBO DNA-free^TM^ Kit (Ambion®, Life Technologies Corporation, California, USA) was used for treatment and removal of any contaminating genomic DNA from the extracted RNA samples according to the manufacturer’s instructions. The Transcriptor First Strand cDNA Synthesis Kit (Roche Applied Science, Mannheim, Germany) was used to synthesise cDNA according to the manufacturer’s instructions. Each reaction tube contained 600 ng RNA, 2.5 μM Oligo (dT), 60 μM random hexamers, 8 mM RT reaction buffer, 1 mM dNTP, 10 U reverse transcriptase, 20 U RNase inhibitor and 6 μL RNAse-DNAse free water to make a total volume of 20 μL. The reactions were performed with an initial stage of 25°C for 10 min, followed by 55°C for 30 min, 85°C for 5 min, and then chilled at 4°C using an Applied Biosystems® Veriti® Thermal Cycler (Applied Biosystems, Life Technologies Corp., Carlsbad CA, USA). Samples were immediately stored at -80°C for later analysis.

### Primer design

All primers were designed using the National Centre for Biotechnology Information (NCBI), primer Basic Local Alignment Search Tool (BLAST) (Bethesda, Maryland, USA) (http://www.ncbi.nlm.nih.gov/nucleotide).

The primers were designed to have the following features: PCR product of less than 150 bp, primers must span an exon-exon junction, show no complementarity to extraneous targets, have minimal primer-dimer and primer hairpin formation, and similar melting temperature and guanine-cytosine content (GC content %). The best primer set was selected and the PCR amplicon sequence tested for secondary structures at 60°C using the mFold program (http://mfold.rit.albany.edu/?q=mfold). If there were no hairpin loops in the primer binding area, then the primer pair was selected. If there was hairpin loop formation, the entire process was repeated until all conditions were satisfied. Primer sequences are listed in Tables 1–3.

**Table 1:** Ovine kidney genes—transient receptor potential cation channel subfamily V member 5 (TRPV5), transient receptor potential cation channel subfamily V member 6 (TRPV6), Calbindin D_9_k (calD_9k_), Calbindin D_28_k (calD_28k_), plasma membrane calcium ATPase (PMCA), sodium calcium exchanger 1 (NCX1), cytochrome P450 family 27 subfamily B polypeptide 1 (CYP27B1), cytochrome P450 family 24 subfamily A polypeptide 1 (CYP24A1), and vitamin D receptor (VDR) in ovine kidney.

**Gene**	**Full gene name**	**GeneBank (Accession code)**	**Primer (5’-3’)**	**Amplicon length (bp)**	**Primer concentration**	**PCR Efficiency**	**Regression coefficient (R**^**2**^**)**
**TRPV5**	Transient receptor potential cation channel subfamily V member 5	XM_004008320.1	F: CGGGTCAGCAATCATCCTAT	108	250:250 nM	110.6%	0.96
			R: ATTGTGATGACGTGGAATGG				
**TRPV6**	Transient receptor potential cation channel subfamily V member 6	EU310242.1	F: TGATGCTGGAGAAGAAGCTG	118	250:250 nM	93.9%	0.98
			R: TGGTTGATGTCCTGTTTCTCTT				
**calD_9k_**	Calbindin D9k	NC_019484.1	F: TCACTGCTGAACGGCCAGGACA	122	250:250 nM	99.3%	0.99
			R: AGCTCCTCCTTGGACAGTTGGT				
**calD_28k_**	Calbindin D28k	NC_019466.1	F: GCTGGAAAAAGCAAACAAGACTGTTGA	139	400:400 nM	90.8%	0.98
			R: TTCTCCTGCACGGGTAGTAATCTGG				
**PMCA**	Plasma membrane calcium ATPase	NC_019460.1	F: TGCAGCCATAGTATCATTGGGCCT	128	250:250 nM	94.9%	0.99
			R: TTGCCGCTCCTTCAATCCAACCA				
**NCX1**	Sodium calcium exchanger 1	NC_019460.1	F: TGGCGAACATCAACCCGTGCT	93	300:300 nM	98.4%	0.99
			R: TGCAGATTGTAGCGTCGCATCTCG				
**CYP27B1**	Cytochrome P450 family 27 subfamily B polypeptide 1	XM_004006519.1	F: GCAGAGCTTGAGTTGCACAT	119	250:250 nM	102.2%	0.92
			R: CTTCTCTCAGGCACCAGGAC				
**CYP24A1**	Cytochrome P450 family 24 subfamily A polypeptide 1	NC_019470.1	F: CTGTGATGAGAGAGGCCGCATTGA	128	600:600 nM	103.9%	0.99
			R: AGCTTCCTCCCCTGCCTTCTT				
**VDR**	Vitamin D receptor	NC_019460.1	F: TCATGCTGCGCTCCAACCAGT	140	400:400 nM	93.5%	0.98
			R: TGGAACTTGATGAGGGGCTCGAT				
**SDHA**	Succinate dehydrogenase complex	NC_019460.1	F: ACCTGATGCTTTGTGCTCTGC	126	300:300 nM	97.05%	0.99
			R: CCTGGATGGGCTTGGAGTAA				
**PGK1**	Phosphoglycerate kinase 1	NC_019460.1	F: ACTCCTTGCAGCCAGTTGCT	101	300:300 nM	94.5%	0.99
			R: AGCACAAGCCTTCTCCACTTCT				

**Table 2:** Canine kidney genes- transient receptor potential cation channel subfamily V member 5 (TRPV5), transient receptor potential cation channel subfamily V member 6 (TRPV6), Calbindin D_9k_ (calD_9k_), Calbindin D_28k_ (calD_28k_), plasma membrane calcium ATPase (PMCA), sodium calcium exchanger 1 (NCX1), cytochrome P450 family 27 subfamily B polypeptide 1 (CYP27B1), cytochrome P450 family 24 subfamily A polypeptide 1 (CYP24A1), and vitamin D receptor (VDR) in canine kidney.

**Gene**	**Full gene name**	**GeneBank (Accession code)**	**Primer (5’-3’)**	**Amplicon length (bp)**	**Primer concentration**	**PCR Efficiency**	**Regression coefficient (R**^**2**^**)**
**TRPV5**	Transient receptor potential cation channel subfamily V member 5	XM_003639556.2	F: CCACATGCTGCAACAGAAGA	108	500:500 nM	92.7%	0.99
			R: AAGTCACAGTTCCGGTCCAG				
**TRPV6**	Transient receptor potential cation channel subfamily V member 6	XM_539861.5	F: AGAGCCGAGATGAGCAGAAC	98	500:500 nM	102.3%	0.98
			R: CTTGCTGAGAGCCTGGACAT				
**calD_9k_**	Calbindin D9k	XM_843973.2	F: TCTTCTAGCTGCCTTGCTG	104	900:900 nM	101.7%	0.99
			R: CTTCTTTGGCTGCGTATTTT				
**calD_28k_**	Calbindin D28k	XM_848991.4	F: CAGGGAATCAAAATGTGTGG	107	800:800 nM	90.1%	0.99
			R: TCCTTCAGTAAAGCATCCAGTTC				
**PMCA**	Plasma membrane calcium ATPase	XM_005628823.1	F: TGAAAGCTCATTGACTGGTGA	103	250:250 nM	91.7%	0.99
			R: CATTCTTCCAGAGCCTTCCA				
**NCX1**	Sodium calcium exchanger 1	XM_005630379.1	F: CACCATCGGCTTGAAAGATT	114	350:350 nM	104.3%	0.98
			R: GCGTCTGCATACTGATCCTG				
**CYP27B1**	Cytochrome P450 family 27 subfamily B polypeptide 1	XM_538254.4	F: GAGCTGCAAATGGCTTTGGCTCAG	123	300:300 nM	89.2%	0.99
			R: CTGTAGGTTGATGCTCCTCTCGGG				
**CYP24A1**	Cytochrome P450 family 24 subfamily A polypeptide 1	XM_543059.3	F: GAGGCCGCATTGAAGACTTA	102	450:450 nM	94.7%	0.98
			R: CATTCTTCCGAAGGAGTCCA				
**VDR**	Vitamin D receptor	XM_005636920.1	F: AGCATCCAAAAGGTCATTGG	92	900:800 nM	95.2%	0.97
			R: GCACTTGATTTCAGCAGCAC				
**RPL32^‡^**	Ribosomal protein L32	XM_848016.1	F: TGGTTACAGGAGCAACAAGAAA	100	900:900 nM	98.53%	0.99
			R: GCACATCAGCAGCACTTCA				
**ß-actin^‡^**	Beta-actin	AF021873	F: CCAGCAAGGATGAAGATCAAG	100	900:900 nM	102.6%	0.98
			R: TCTGCTGGAAGGTGGACAG				

‡ Peters *et al*., 2007

**Table 3:** Equine kidney genes- transient receptor potential cation channel subfamily V member 5 (TRPV5), transient receptor potential cation channel subfamily V member 6 (TRPV6), Calbindin D_9k_ (calD_9k_), Calbindin D_28k_ (calD_28k_), plasma membrane calcium ATPase (PMCA), sodium calcium exchanger 1 (NCX1), cytochrome P450 family 27 subfamily B polypeptide 1 (CYP27B1), cytochrome P450 family 24 subfamily A polypeptide 1 (CYP24A1), and vitamin D receptor (VDR) in equine kidney.

**Gene**	**Full gene name**	**GeneBank (accession code)**	**Primer (5’-3’)**	**Amplicon length (bp)**	**Primer concentration**	**PCR Efficiency**	**Regression coefficient (R2)**
**TRPV5**	Transient receptor potential cation channel subfamily V member 5	AY944068	F: ACACTGTGATGTTCCAGCACCTGA	102	75:75 nM	96.9%	0.99
			R: AGGAGTCGATCTCTGTGAGGTCAT				
**TRPV6**	Transient receptor potential cation channel subfamily V member 6	XM_001490905.2	F: CCTCAAGCCCATCACCAGTA	97	250:250 nM	104.5%	0.97
			R: GCCATCCTTAGGGGTCAAGT				
**calD_9k_**	Calbindin D9k	AY229893	F: GCGTGAAAAAGTCTCCTGAA	218	700:700 nM	97.08%	0.99
			R: TCACTAACACCTGGAATTCTTCAA				
**calD_28k_**	Calbindin D28k	NM_001163952	F: ACGGCTTGGTCTTCCTTGACAG	101	75:100 nM	102.7%	0.99
			R: TGCGTGTGTGAGTATGTGTGAGTG				
**PMCA**	Plasma membrane calcium ATPase	DQ160196	F: AAACGCCGCCGATAGTGCAAATAC	182	200:200 nM	102.7%	0.99
			R: CCTTCTTGTGCATGTTGGCCTTCTTC				
**NCX1**	Sodium calcium exchanger 1	DQ178640	F: TGGCCATAACTTTACTGCGGGAGA	100	500:250 nM	92.4%	0.99
			R: GGACCACATAAACACAAAGCGCGA				
**CYP27B1**	Cytochrome P450 family 27 subfamily B polypeptide 1	NM_001163957.1	F: CAGAGACATTCATGTGGGTGA	117	300:300 nM	93.9%	0.98
			R: GCTGGACGAAAAGAATTTGG				
**CYP24A1**	Cytochrome P450 family 24 subfamily A polypeptide 1	XM_003363957.2	F: GTGTGATGAAAGAGGCCACATTGA	113	350:350 nM	91.6%	0.99
			R: CGTTCTGCTGGAGGAGCCCG				
**VDR**	Vitamin D receptor	XM_005611070.1	F: ACAGCATCCAAAAGGTGGTC	89	500:500 nM	99.9%	0.99
			R: TGACTTCAGCAGCACGATCT				
**YWHAZ^₠^**	Zeta polypeptide	XM_001492988.3	F: TGTTGTAGGAGCCCGTAGGT	95	300:300 nM	93.4%	0.99
			R: ATTCTCGAGCCATCTGCTGT				
**HPRT1**	Hypoxanthine phosphoribosyltransferase 1	AY372182	F: TTGCTGACCTGCTGGATTAT	120	500:500 nM	93.8%	0.99
			R: TTATGTCCCCTGTTGACTGGT				

₠ Kayis *et al*., 2011

### Real-time qPCR

Real-time qPCR was performed using the StepOne Plus real-time PCR machine (Applied Biosystems, Life Technologies Corp., Carlsbad CA, USA). The primer concentrations determined to be optimal for each primer pair PCR are shown in Tables 1–3. Real-time PCR reactions (10 μL) contained 5 μL Fast SYBR Green real-time PCR Master Mix (Applied Biosystems, Life Technologies Corp., Carlsbad CA, USA), the primer pair at concentrations given in Tables 1–3, 10 ng of cDNA template, and RNAase-DNAse free water. The PCR protocol consisted of a denaturation step at 95°C for 20 s, followed by 40 cycles at 95°C for 3 s and 60°C for 30 s; ending with a melt curve ranging from 60°C to 95°C with a heating rate of 0.3°C/15 s. Negative controls of water and reaction mix without reverse transcriptase were included in every PCR run and all samples were run in duplicate. Standard curves were produced for each target to determine the accuracy (R^2^ ≥0.98) and efficiency (90–110%) of the real-time PCR reactions (Tables 1–3). The real-time data were analysed using the StepOne plus software (Applied Biosystems, Life Technologies Corp., Carlsbad CA, USA) and then exported into an Excel datasheet (Microsoft Excel 2010) for further analysis.

All samples were normalised relative to the expression of appropriate housekeeping genes (HKGs) as follow: zeta polypeptide (YWHAZ) [12] and hypoxanthine phosphoribosyltransferase 1 (HPRT1) in horses (paper submitted for publication), ribosomal protein L32 (RPL32) and beta-actin (ß-actin) in dogs [13], and succinate dehydrogenase complex (SDHA) and phosphoglycerate kinase 1 (PGK1) in sheep (S1 Table, S1 and S2 Figs).

### Statistical analysis of the RT-qPCR data

Expression of nine calcitropic genes (TRPV5, TRPV6, calD_9k_, calD_28k_, PMCA, NCX1, CYP27B1, CYP24A1 and VDR) was evaluated in 10 ovine, five canine and 10 equine kidney samples. The Ct (cycle threshold) values from RT-qPCR runs were exported into an Excel datasheet (Microsoft Excel 2010). The Ct values of each gene in each animal were normalised with the species related HKGs using:

$$Ĉt_{ij}=Ct_{ij}-\bar{Rt_{j}}+\bar{Rt}$$

where *Ct_ij_* is the level of expression of gene *i* for sample *j*, $\bar{Rt_{j}}$ is the mean expression of sample *j* on the HKGs, and $\bar{Rt}$ is the mean expression on the HKGs across all samples [14]. The expression per gene was estimated using a linear mixed effects model including a random effect accounting for the two replicates per sample. The model equation was:

$$Ĉt_{ijk}=Gene_{i}+\mu_{j}+\epsilon_{ijk}$$

where $Ĉt_{ijk}$ is the normalised level of expression of gene *i* for sample *j* and replicate *k*, *Gene_i_* is the average expression for gene *i*, *μ_j_* is a random effect for sample, and *ϵ_ijk_* are the residuals. Mean expression levels and 95% confidence intervals were computed by simulating from the fitted model, accounting for the variation due to the per-sample random effects. Spearman’s pairwise correlation and scatterplots were used to study the correlation between normalised expression across different genes.

All statistical models and plots were produced in R [15]. The mixed effects model was fit using the lme4 package for R [16].

## Results

**Fig 1:**
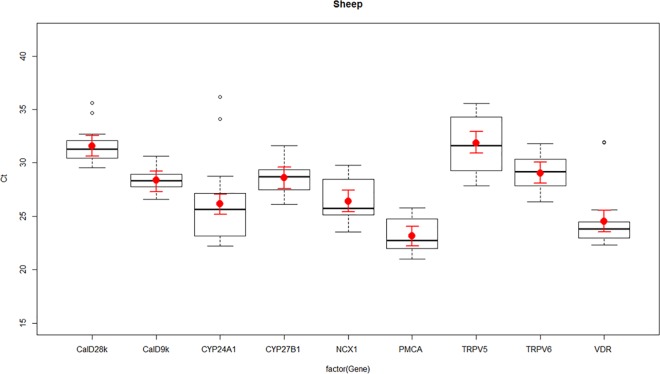
Expression of vitamin D and calcium channel transcripts in ovine kidney—the transient receptor potential cation channel subfamily V member 5 (TRPV5), the transient receptor potential cation channel subfamily V member 6 (TRPV6), Calbindin D_9_k (calD_9k_), Calbindin D_28_k (calD_28k_), plasma membrane calcium ATPase (PMCA), sodium calcium exchanger 1 (NCX1), cytochrome P450 family 27 subfamily B polypeptide 1 (CYP27B1), cytochrome P450 family 24 subfamily A polypeptide 1 (CYP24A1), and vitamin D receptor (VDR) in ovine kidney as determined by RT-qPCR. Boxplots represent normalised cycle threshold numbers (Ct values), where the median expression levels of genes are presented as black bars, the average expression of genes are presented as red dots, and red line bars represent 95% confidence interval (CI) of genes, accounting for replicates.

**Fig 2:**
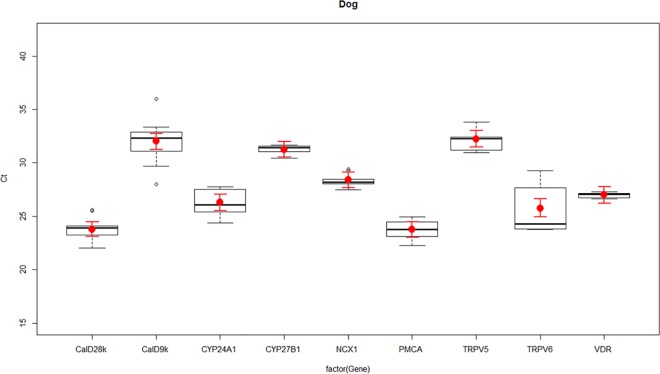
Expression of vitamin D and calcium channel transcripts in canine kidney—the transient receptor potential cation channel subfamily V member 5 (TRPV5), the transient receptor potential cation channel subfamily V member 6 (TRPV6), Calbindin D_9_k (calD_9k_), Calbindin D_28_k (calD_28k_), plasma membrane calcium ATPase (PMCA), sodium calcium exchanger 1 (NCX1), cytochrome P450 family 27 subfamily B polypeptide 1 (CYP27B1), cytochrome P450 family 24 subfamily A polypeptide 1 (CYP24A1), and vitamin D receptor (VDR) in dog kidney as determined by RT-qPCR. Boxplots represent normalised cycle threshold numbers (Ct values), where the median expression levels of genes are presented as black bars, the average expression of genes are presented as red dots, and red line bars represent 95% confidence interval (CI) of genes, accounting for replicates.

**Fig 3:**
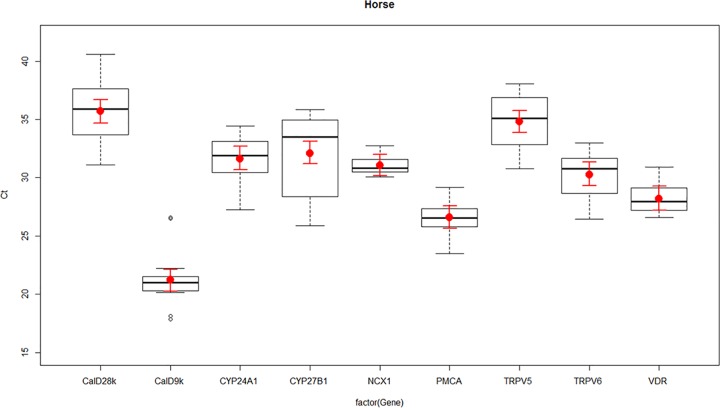
Expression of vitamin D and calcium channel transcripts in equine—the transient receptor potential cation channel subfamily V member 5 (TRPV5), the transient receptor potential cation channel subfamily V member 6 (TRPV6), Calbindin D_9_k (calD_9k_), Calbindin D_28_k (calD_28k_), plasma membrane calcium ATPase (PMCA), sodium calcium exchanger 1 (NCX1), cytochrome P450 family 27 subfamily B polypeptide 1 (CYP27B1), cytochrome P450 family 24 subfamily A polypeptide 1 (CYP24A1), and vitamin D receptor (VDR)in equine kidney as determined by RT-qPCR. Boxplots represent normalised cycle threshold numbers (Ct values), where the median expression levels of genes are presented as black bars, the average expression of genes are presented as red dots, and red line bars represent 95% confidence interval (CI) of genes, accounting for replicates.

**Fig 4:**
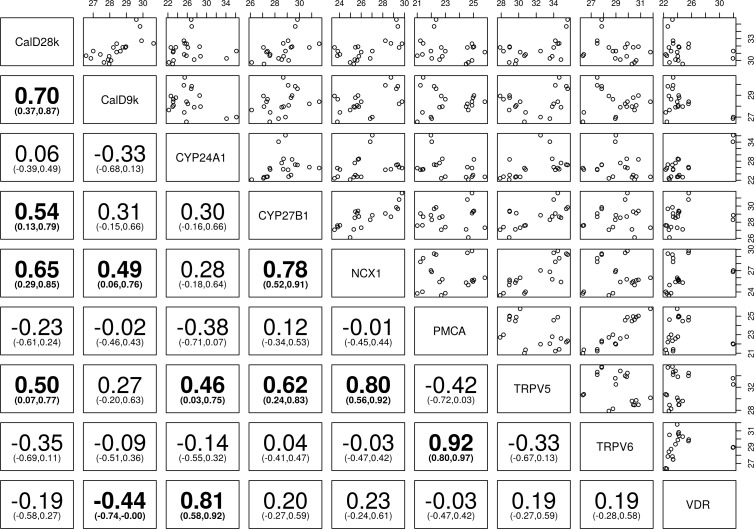
Correlation of vitamin D and calcium channels transcripts in ovine kidney—Spearman’s correlation (95% Confidence Intervals) between the transient receptor potential cation channel subfamily V member 5 (TRPV5), the transient receptor potential cation channel subfamily V member 6 (TRPV6), calbindin D_9_k (calD_9k_), calbindin D_28_k (calD_28k_), plasma membrane calcium ATPase (PMCA), sodium calcium exchanger 1 (NCX1), cytochrome P450 family 27 subfamily B polypeptide 1 (CYP27B1), cytochrome P450 family 24 subfamily A polypeptide 1 (CYP24A1), and vitamin D receptor (VDR). Bolded numbers indicate statistically significant positive and/or negative correlation of different genes towards each other (P< 0.05), the number in parenthesis represent 95% confidence interval (CI), the numbers on the top and right hand side represent normalised level of expression of gene.

**Fig 5:**
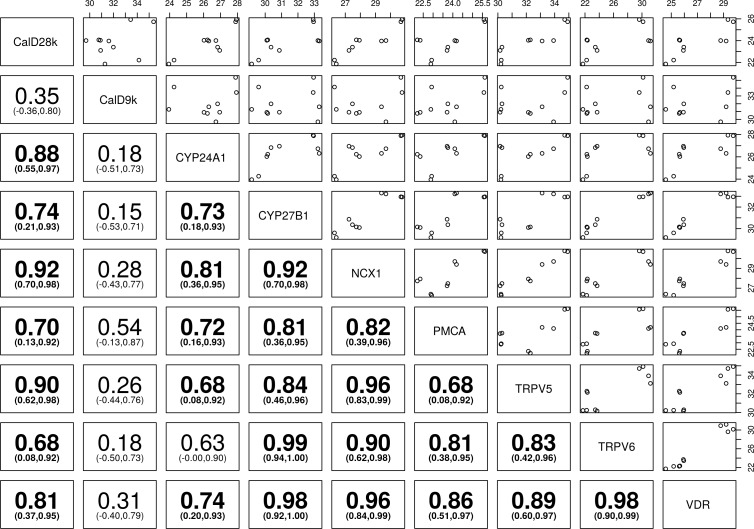
Correlation of vitamin D and calcium channels transcripts in canine kidney—Spearman’s correlation (95% Confidence Intervals) between the transient receptor potential cation channel subfamily V member 5 (TRPV5), the transient receptor potential cation channel subfamily V member 6 (TRPV6), calbindin D_9_k (calD_9k_), calbindin D_28_k (calD_28k_), plasma membrane calcium ATPase (PMCA), sodium calcium exchanger 1 (NCX1), cytochrome P450 family 27 subfamily B polypeptide 1 (CYP27B1), cytochrome P450 family 24 subfamily A polypeptide 1 (CYP24A1), and vitamin D receptor (VDR). Bolded numbers indicate statistically significant positive and/or negative correlation of different genes towards each other (P< 0.05), the number in parenthesis represent 95% confidence interval (CI), the numbers on the top and right hand side represent normalised level of expression of gene.

**Fig 6:**
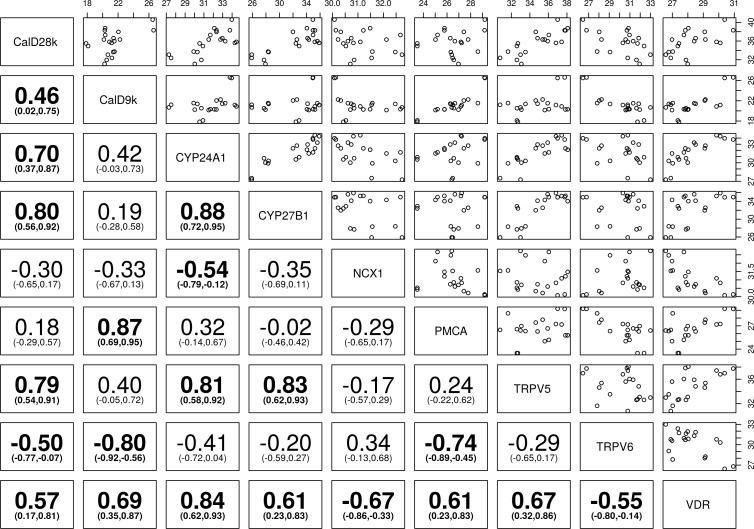
Correlation of vitamin D and calcium channels transcripts in equine kidney—Spearman’s correlation (95% Confidence Intervals) between the transient receptor potential cation channel subfamily V member 5 (TRPV5), the transient receptor potential cation channel subfamily V member 6 (TRPV6), calbindin D_9_k (calD_9k_), calbindin D_28_k (calD_28k_), plasma membrane calcium ATPase (PMCA), sodium calcium exchanger 1 (NCX1), cytochrome P450 family 27 subfamily B polypeptide 1 (CYP27B1), cytochrome P450 family 24 subfamily A polypeptide 1 (CYP24A1), and vitamin D receptor (VDR). Bolded numbers indicate statistically significant positive and/or negative correlation of different genes towards each other (P< 0.05), the number in parenthesis represent 95% confidence interval (CI), the numbers on the top and right hand side represent normalised level of expression of gene.

Tissue-specific mRNA expression of TRPV5, TRPV6, calD_9k_, calD_28k_, PMCA, NCX1, CYP27B1, CYP24A1, and VDR in the sheep, dog and horse kidney was analysed by RT-qPCR, and all target genes were detected (Figs 1–6). Sequencing analysis showed all PCR products had 100% identity to the target genes. Low Ct values equal high expression.

### Comparison of the expression of calcium and vitamin D responsive genes in ovine, canine and equine kidney

Differences in the normalised expression levels of calcium channels and vitamin D responsive genes TRPV5, TRPV6, calD_9k_, calD_28k_, PMCA, NCX1, CYP27B1, CYP24A1 and VDR were identified between ovine, canine and equine kidney, presented in Figs 1–3.

TRPV5 and TRPV6 had similar expression patterns in sheep, dogs and horses, with TRPV6 having greater expression than TRPV5. CalD_9k_ and calD_28k_ were both expressed in the ovine, canine and equine kidney. The difference in expression between calD_28k_ and calD_9k_ appeared to be greater in horses than sheep but the pattern was similar, in that expression levels of calD_28k_ were lower than calD_9k_ in both sheep and horses. In dogs, calD_9k_ had lower expression than calD_28k_ (Figs 1–3).

Ovine, canine and equine kidney showed similar patterns of NCX1 and PMCA expression, where the expression levels of NCX1 were always lower than PMCA. Differences in expression between CYP27B1 and CYP24A1 were particularly obvious between species, where horses showed very similar levels of expression for CYP27B1 and CYP24A1, while in sheep and dogs CYP27B1 expression was lower than CYP24A1. VDR had a similar high level of expression in all species (Figs 1–3).

The RT-qPCR data were analysed and plotted to determine the pairwise correlation of vitamin D responsive genes with each other in the sheep, dog and horse kidney, presented in Figs 4–6.

CalD_28K_ showed significant positive correlation with NCX1 and CYP24A1 in dogs, CYP27B1, TRPV5, and CYP24A1 in horses, and NCX1, CYP27B1, and TRPV5 in sheep (*P*< 0.05). CalD_9k_ showed significant positive correlation with PMCA in dogs and horses (*P*<0.05), whereas strong positive correlation with NCX1 and strong negative correlation with VDR was present in sheep. CalD_28k_ and calD_9k_ were positively correlated in sheep and horses (Figs 4–6).

TRPV6 and PMCA showed significant positive correlation in sheep, whereas significant negative correlation was seen in dogs and horses (*P*<0.05). PMCA showed significant negative correlation with VDR in dogs, compared with significant positive correlation with VDR in horses (*P*< 0.05). TRPV6 showed significant negative correlation with VDR in equine kidney (*P*< 0.05). NCX1 showed significant positive correlation with TRPV5 in dogs, and significant negative correlation with VDR in horses (*P*< 0.05) (Figs 4–6).

CYP27B1 showed significant positive correlation with NCX1 in sheep (*P*< 0.05), but its expression was not significantly correlated with other genes in dogs. CYP24A1 and CYP27B1 were significantly positively correlated with TRPV5 in sheep, and TRPV5 and VDR in horses (*P*< 0.05). CYP24A1 and CYP27B1 were significantly positively correlated in equine kidney (*P*< 0.05) (Figs 4–6).

## Discussion

To our knowledge this is the first investigation describing and comparing the expression of the vitamin D responsive genes involved in renal calcium transport in sheep, dogs and horses. Together these genes, TRPV5, TRPV6, calD_9k_, calD_28k_, PMCA, NCX1, CYP27B1, CYP24A1, and VDR regulate transcellular calcium transport in epithelial cells [3, 11]. Different studies published on calcium related genes in mice [17], humans [18, 19], sheep [20–23], rabbits [24], goats [21–23], horses [25–27], and dogs [28, 29], demonstrated a correlation between the expression of calcium transport genes and the capacity of cells for calcium absorption [24]. Therefore, gene function is strongly related to their location and magnitude of expression.

The results of this study showed that TRPV5, TRPV6, calD_9k_, calD_28k_, PMCA, NCX1, CYP27B1, CYP24A1 and VDR mRNAs were detectable in the kidney of sheep, dogs and horses although, the expression levels of each gene was different in each species. The nine genes investigated can be divided into five groups based on their function in calcium metabolism. The two selective calcium channels, TRPV5 and TRPV6, mediate apical influx of calcium into epithelial cells of the renal tubules [1]. CalD_9k_ and calD_28k_, as vitamin D-dependent calcium-binding proteins, mediate the diffusion of calcium through the basolateral membrane [3], while PMCA and NCX1 mediate the secretion of calcium across the basolateral membrane [30]. These calcium channels and calcium binding proteins are vitamin D responsive genes, therefore 1,25(OH)_2_D initiates its biological effect in gene expression via binding to VDR [10]. VDR interacts with the retinoid X receptor (RXR) to form a heterodimer receptor complex, VDR-RXR, which binds to vitamin D responsive elements in the region of genes directly controlled by 1,25(OH)_2_D and alters gene expression [31]. CYP27B1 and CYP24A1 play a critical role in governing plasma concentration of 1,25(OH)_2_D through activation and degradation of vitamin D [32].

Mammalian transient receptor potential channels (TRPs) are cation-permeable channels involved in a variety of physiological processes and are categorised into six different subgroups, including TRPV channels [33]. The most calcium selective channels within the TRPV subgroup are TRPV5 and TRPV6, which are closely related and considered the gatekeepers of transcellular calcium transport [1]. Although TRPV5 and TRPV6 are very similar from an electrophysiological point of view and share many functional properties, including calcium dependent inactivation and regulation by 1,25(OH)_2_D and calcium [34], some significant differences were revealed through detailed comparison of the N- and C-termini of these channels. For instance, TRPV5 channels are approximately 100 times more sensitive to the potent channel blocker ruthenium red than TRPV6 channels, the kinetic differences between calcium (Ca^2+^) and barium (Ba^2+^) currents are more pronounced for TRPV6 than for TRPV5, and TRPV6 has faster initial inactivation than TRPV5 [35]. It is suggested that TRPV5 is involved in transcellular calcium reabsorption in the kidney and predominantly expressed in the distal convoluted (DCT) and connecting tubule (CNT), whereas TRPV6 regulates and increases calcium transport in the intestine [36]. Our findings showed that both TRPV5 and TRPV6 are expressed in ovine, canine and equine kidney, which is in agreement with previous reports [20, 25, 26, 28, 29]. However, the high renal expression of TRPV6 compared to TRPV5 was an unexpected finding, since TRPV5 is thought to be the main renal and TRPV6 the main intestinal apical calcium channel in mammals [37]. TRPV6 has quicker calcium dependent inactivation and slower recovery from inactivation than TRPV5 [35], therefore greater expression of this gene may be needed in the tissue to compensate for this and regulate calcium transport. While TRPV5 and TRPV6 are the principal targets of calcitropic hormones, extracellular and intracellular signalling by plasma calcium concentration and associated proteins play important roles in their regulation [38]. There was limited information on the dietary regimen of the animals used in the present study, including the dietary content of calcium, phosphorus and other minerals, however, the results do indicate that TRPV6 acts cooperatively with TRPV5 in the kidney and plays a substantial role in renal calcium reabsorption.

Calbindin proteins are specialised calcium buffering proteins involved in facilitating different steps of epithelial calcium transport and control a continuous in-flow of intracellular calcium from the TRPV5 and TRPV6 channels [1, 3, 38]. The findings in this study indicate that calD_28K_ and calD_9K_ are co-expressed in the kidney, suggesting that calD_9K_ acts cooperatively with calD_28K_ and has an important role in calcium reabsorption in the kidney of sheep, horse and dog. The expression patterns of calD_28K_ mRNA in ovine, canine and equine kidney have been previously studied [25, 28, 39], although to the authors’ knowledge there are no studies performed on calD_9K_ in ovine and canine kidney. The only previously published study on calD_9K_ in equine kidney reported that the expression of calD_28k_ was much higher in the horse kidney than calD_9k_ [25], in contrast with the present findings. The horses used in the current study were older than horses used in the previous study, which may explain the differences as calcium demands are higher in younger horses. In the study by Rourke *et al*., 2010, calD_28k_ expression was detected primarily in the kidney and calD_9K_ in the intestine, however, the results of the current study indicated that calD_9K_ expression was significantly higher than calD_28K_ in ovine and equine kidney but not in canine kidney. This may be associated with the nutritional alkali load in grazing animals (sheep and horses) compared with the acid load in carnivores (dogs), based on the nature of their diet. Urine composition is directly related to the animals’ diet, leading to alkaline urine (pH 7.0–8.0) in herbivores and more acidic urine in carnivores (pH 5.5–7.0) [40]. Different dietary sources, along with our finding, might suggest calD_9K_ is the specialised calcium buffering protein for calcium transport in grazing animals, whereas calD_28K_ is the specialised form in carnivores.

The extracellular concentration of calcium is much greater than the intracellular concentration and calcium cannot exit cells by diffusion. The extrusion of calcium across the basolateral membrane into the peritubular fluid is mediated by PMCA [41] and/or NCX1 [42]. PMCA is powered by ATP, where one molecule of ATP is hydrolysed to export each calcium ion, while NCX1 removes a calcium ion in exchange for three sodium ions entering the cell [30]. PMCA has a high affinity and low capacity towards calcium, therefore it binds tightly to calcium even when the concentration of calcium in the cell is low, but the removal rate of calcium from the cell through the PMCA pump is slow [43]. In contrast, NCX1 has a low affinity and high capacity towards calcium, thus it does not bind tightly to calcium and is capable of transporting and removing large amounts of calcium rapidly [30]. PMCA and NCX1 are expressed in many different tissues including the kidney, and their activities complement each other [30]. Our findings showed high expression of PMCA and NCX1 mRNA, suggesting the co-localization of NCX1 and PMCA in ovine, canine and equine kidney, which is in agreement with the previous studies in dogs and horses [25, 26, 28, 29]. We found that the most dominant channel with relatively higher expression in the kidney was PMCA mRNA, which fits with the high expression of TRPV6 mRNA and suggests slow transport of calcium is predominantly used in the kidney of the animals studied. These findings suggest that TRPV6 and PMCA are expressed more for slow continuous transepithelial calcium transport in the kidney with TRPV5 and NCX1, as fast transepithelial calcium transport, perhaps being expressed as required to rapidly remove large amount of calcium from the cell. Another possible explanation could be the animals being in a state of normocalcemia at the time of sampling.

When the correlation of genes was examined it became clear that TRPV6, calD_9k_ and/or calD_28k_, and PMCA transcripts were significantly correlated (*P*< 0.05) in ovine, canine and equine kidney. Therefore, it can be suggested that this group of genes control the rate of entry, transport and removal of calcium in renal epithelial cells.

Urine composition and the concentration of calcium are different in mammalian species, with a direct link to their diet. For instance, reported fractional urinary clearances for calcium (FE_Ca_%) in sheep range from 0.010–8.90, in dogs from 0.04–5.6, and in horses from 1.3–33.0 [44]. Horses have higher urinary excretion of calcium, decreased parathyroid gland sensitivity to calcium and lower serum 1,25(OH)_2_D concentration compared with sheep and dogs [45–48]. In equine kidney, CYP27B1 and CYP24A1 transcripts had very similar expression levels, while in sheep and dogs expression of CYP24A1 was higher than CYP27B1. This suggests that in horses the rate of activation and degradation of vitamin D (1,25(OH)_2_D) occurs in a parallel manner and that 1,25(OH)_2_D production is tightly controlled, while in sheep and dogs degradation of 1,25(OH)_2_D outstrips production. However, in sheep and dogs there was no consistently significant correlation of the calcium transporting genes with the VDR, whereas in horses all the calcium transport transcripts were significantly correlated with VDR mRNA. This finding may suggest that even with the very low serum concentration of 1,25(OH)_2_D in horses compared to other animals, it does closely and tightly regulate these vitamin D-responsive calcium transport genes in the kidney. It does however not explain the high excretion of calcium in equine urine, perhaps it is unrelated to vitamin D and the decreased sensitivity of the parathyroid gland to calcium in the horse is the primary mechanism, but further research is required to determine this.

The current study has some limitations and it is not possible to draw firm conclusions regarding transepithelial calcium transport in the kidney without information on serum biochemistry and the dietary history of the animals. In addition, RT-qPCR performed in this study is a snapshot in time of calcium homeostasis in the animal, and factors such as age, diet, and life history will alter gene expression.

The correlated expression patterns of TRPV6, calD_9k_ /calD_28k_, and PMCA mRNA detected in the ovine, canine and equine kidney emphasise the importance of these genes in active transepithelial calcium transport in the kidney of these species. In equine kidney, all vitamin D-responsive and calcium transporting transcripts were highly correlated with VDR, whereas sheep and dogs did not show such high correlation, indicating the important regulatory function of VDR in the active renal transepithelial calcium transport in horses. The renal expression pattern and correlation of CYP27B1 and CYP24A1 mRNA in horses were particularly different to those in sheep and dogs suggesting that despite low serum vitamin D concentrations, vitamin D still plays a significant role in calcium metabolism in horses. The strong co-expression of renal vitamin D-responsive transcripts suggests that these genes work in harmony to facilitate the transepithelial calcium transport in ovine, canine and equine kidney.

## Supporting Information

S1 Fig Gene expression stability of the candidate housekeeping genes in ovine renal tissue analysed by the geNorm program. **Lower M values correspond to the most stable and most suitable HKGs for normalisation.** The HKGs expression stability data were analysed with geNorm (qbase+ 3.0, Biogazelle, Zwijnaarde, Belgium) (Vandesompele *et al*., 2002; Hellemans *et al*., 2007); this program generates a measure of HKGs stability, which can be used to rank the HKGs. M values less than 1.0 and V values less than 0.15 are considered optimal. SDHA and PGK1 genes were ranked as the two most stably expressed HKG in ovine kidney), thus they were selected for normalisation in subsequent qRT-PCR experiments.
(TIF)

S2 Fig Evaluation of the optimum number of housekeeping genes in ovine renal tissue according to the geNorm software. (TIF)

S1 Table Ovine housekeeping gene primer sequence, amplicon length, real time PCR efficiency and regression coefficient. Determination of most stable housekeeping genes used for use with real-time quantitative reverse transcriptase polymerase chain reaction (RT-qPCR) assays in ovine kidney. Real time quantitative reverse transcriptase polymerase chain reaction assays were run as described in the Materials and Methods using the following primer pairs.
(DOCX)
