# Supplementary figures and images for: Evaluation and Comparison of Vitamin D Responsive Gene Expression in Ovine, Canine and Equine Kidney
Source: PLoS One. 2016 Sep 15;11(9):e0162598. doi: 10.1371/journal.pone.0162598 (PMC5025205; doi:10.1371/journal.pone.0162598)

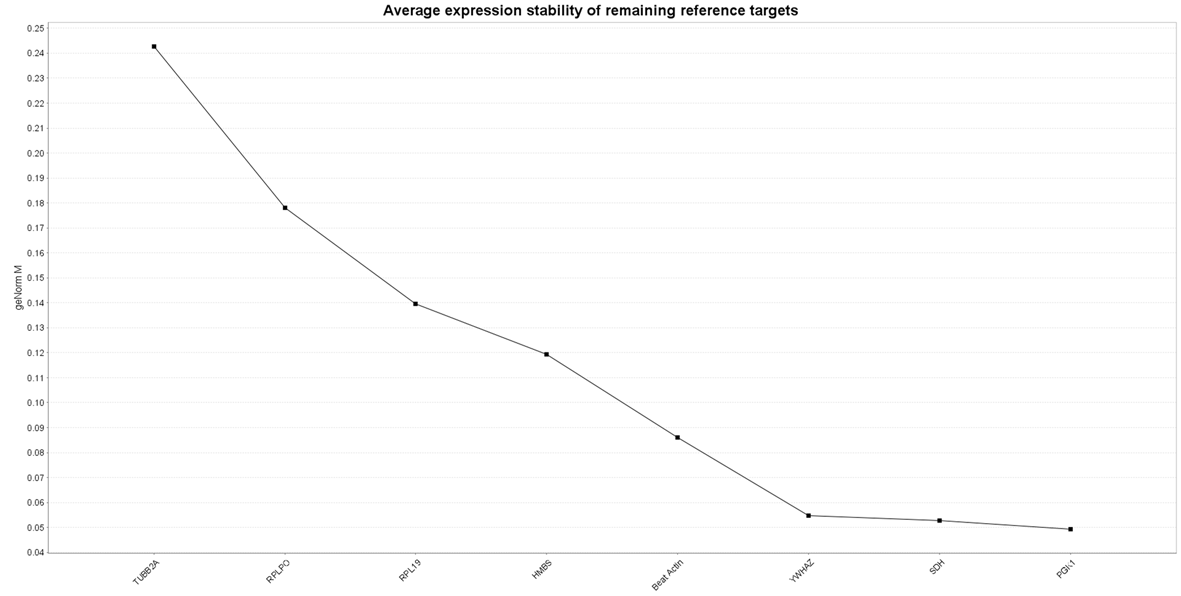

Supplement: S1 Fig — Lower M values correspond to the most stable and most suitable HKGs for normalisation. The HKGs expression stability data were analysed with geNorm (qbase+ 3.0, Biogazelle, Zwijnaarde, Belgium) (Vandesompele et al., 2002; Hellemans et al., 2007); this program generates a measure of HKGs stability, which can be used to rank the HKGs. M values less than 1.0 and V values less than 0.15 are considered optimal. SDHA and PGK1 genes were ranked as the two most stably expressed HKG in ovine kidney), thus they were selected for normalisation in subsequent qRT-PCR experiments. (TIF) [file pone.0162598.s001.tif]

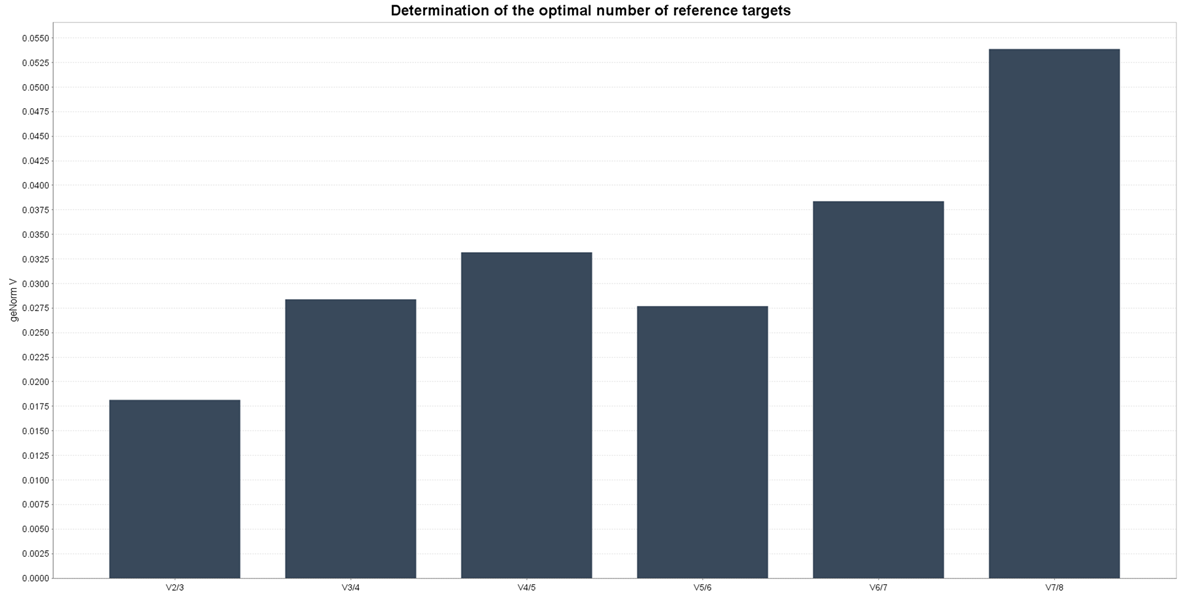

Supplement: S2 Fig — (TIF) [file pone.0162598.s002.tif]
