# Supplementary material for: Evaluation and Comparison of Vitamin D Responsive Gene Expression in Ovine, Canine and Equine Kidney
Source: PLoS One. 2016 Sep 15;11(9):e0162598. doi: 10.1371/journal.pone.0162598 (PMC5025205; doi:10.1371/journal.pone.0162598)
Supplement: S1 Table — Determination of most stable housekeeping genes used for use with real-time quantitative reverse transcriptase polymerase chain reaction (RT-qPCR) assays in ovine kidney. Real time quantitative reverse transcriptase polymerase chain reaction assays were run as described in the Materials and Methods using the following primer pairs. (DOCX) [file pone.0162598.s003.docx]

**S1 Table. Ovine housekeeping gene primer sequence, amplicon length, real time PCR efficiency and regression coefficient.**

| Gene | Full gene name | Primer (5’-3’) | Amplicon length (bp) | Primer concentration | PCR Efficiency | Regression coefficient (R^2^) |
| --- | --- | --- | --- | --- | --- | --- |
| RPLP0 | Ribosomal Protein Lateral Stalk Subunit P0 | F: CAACCCTGAAGTGCTTGACAT | 227 | 300:300 nM | 102.9% | 0.99 |
|  |  | R: AGGCAGATGGATCAGCCA |  |  |  |  |
| TUBB2A | Tubulin Beta 2A Class IIa | F: ACACTGTGGTTGAGCCCTACA | 120 | 300:300 nM | 109.1% | 1 |
|  |  | R: GCTTGAGGGTGCGGAAA |  |  |  |  |
| HMBS | Hydroxymethylbilane Synthase | F: CCTTGGAGAGGAATGAAGTGG | 80 | 300:300 nM | 90.3% | 0.98 |
|  |  | R: AATGGTGAAGCCAGGAGGAA |  |  |  |  |
| RPL19 | Ribosomal Protein L19 | F: AGCCTGTGACTGTCCATTCC | 126 | 300:300 nM | 90.6% | 0.99 |
|  |  | R: ACGTTACCTTCTCGGGCATT |  |  |  |  |
| YWHAZ | Zeta polypeptide | F: AGACGGAAGGTGCTGAGAAA | 123 | 250:250 nM | 92.2% | 0.97 |
|  |  | R: CGTTGGGGATCAAGAACTTT |  |  |  |  |
| ACTB | Beta-actin | F: CCAACCGTGAGAAGATGACC | 97 | 250:250 nM | 91.8% | 0.99 |
|  |  | R: CCAGAGGCGTACAGGGACAG |  |  |  |  |
| SDHA | Succinate dehydrogenase complex | F: ACCTGATGCTTTGTGCTCTGC | 126 | 300:300 nM | 97.05% | 0.99 |
|  |  | R: CCTGGATGGGCTTGGAGTAA |  |  |  |  |
| PGK1 | [Phosphoglycerate kinase 1](https://www.google.co.nz/url?sa=t&rct=j&q=&esrc=s&source=web&cd=1&cad=rja&uact=8&ved=0CCcQFjAAahUKEwiuofPYq8rHAhUDOKYKHbDzCsg&url=http%3A%2F%2Fwww.ncbi.nlm.nih.gov%2Fgene%2F5230&ei=JpPfVa72OIPwmAWw56vADA&usg=AFQjCNFCO1rWWeJrzIeWqP4fZqnzsv_JdA) | F: ACTCCTTGCAGCCAGTTGCT | 101 | 300:300 nM | 94.5% | 0.99 |
|  |  | R: AGCACAAGCCTTCTCCACTTCT |  |  |  |  |
